# Characterizing Ligand-Gated Ion Channel Receptors with Genetically Encoded Ca^++^ Sensors

**DOI:** 10.1371/journal.pone.0016519

**Published:** 2011-01-28

**Authors:** John G. Yamauchi, Ákos Nemecz, Quoc Thang Nguyen, Arnaud Muller, Lee F. Schroeder, Todd T. Talley, Jon Lindstrom, David Kleinfeld, Palmer Taylor

**Affiliations:** 1 Department of Pharmacology, Skaggs School of Pharmacy and Pharmaceutical Sciences, University of California San Diego, La Jolla, California, United States of America; 2 Biomedical Sciences Graduate Program, University of California San Diego, La Jolla, California, United States of America; 3 Department of Chemistry and Biochemistry, University of California San Diego, La Jolla, California, United States of America; 4 Department of Physics, University of California San Diego, La Jolla, California, United States of America; 5 Department of Neurosciences, School of Medicine, University of Pennsylvania, Philadelphia, Pennsylvania, United States of America; National Institute on Aging Intramural Research Program, United States of America

## Abstract

We present a cell based system and experimental approach to characterize agonist and antagonist selectivity for ligand-gated ion channels (LGIC) by developing sensor cells stably expressing a Ca^2+^ permeable LGIC and a genetically encoded Förster (or fluorescence) resonance energy transfer (FRET)-based calcium sensor. In particular, we describe separate lines with human α7 and human α4β2 nicotinic acetylcholine receptors, mouse 5-HT_3A_ serotonin receptors and a chimera of human α7/mouse 5-HT_3A_ receptors. Complete concentration-response curves for agonists and Schild plots of antagonists were generated from these sensors and the results validate known pharmacology of the receptors tested. Concentration-response relations can be generated from either the initial rate or maximal amplitudes of FRET-signal. Although assaying at a medium throughput level, this pharmacological fluorescence detection technique employs a clonal line for stability and has versatility for screening laboratory generated congeners as agonists or antagonists on multiple subtypes of ligand-gated ion channels. The clonal sensor lines are also compatible with *in vivo* usage to measure indirectly receptor activation by endogenous neurotransmitters.

## Introduction

Nicotinic acetylcholine receptors (nAChRs) are pentameric ligand-gated ion channels (LGIC) belonging to the sub-family of Cys-loop receptors. They are found in the central and peripheral nervous systems of both vertebrate and invertebrate species. Specific nAChR subtypes are recognized pharmaceutical targets for many central nervous system (CNS) diseases and conditions [1]. For example: the α7-nAChR is targeted for schizophrenia [2], Alzheimer's disease [3], and cognition enhancement [4], and the α4β2-nAChR is also targeted for Alzheimer's disease [5], [6], and for tobacco addiction [7], [8]. Therefore, a particular challenge in therapeutic considerations is achieving subtype selectivity among the nAChRs. Sorting out the desirable actions within the subunit diversity of the nAChR family from the largely unwanted responses, via off-target receptors, is an important facet of the therapeutic approach [9].

**Figure 1:**
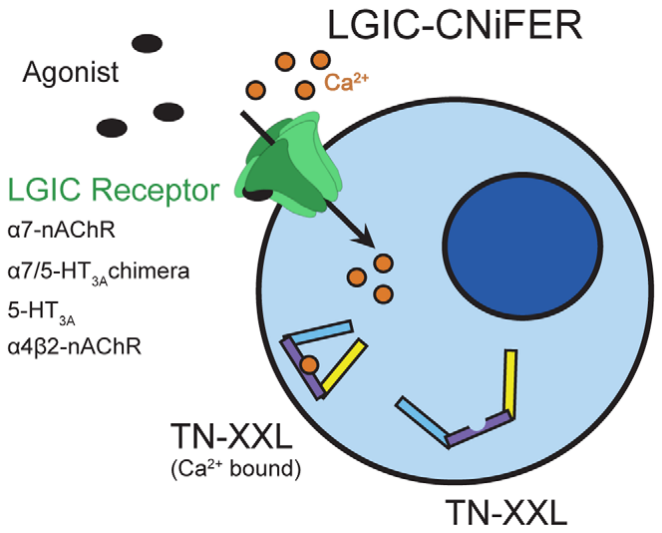
Simplistic representation of LGIC-CNiFERs and their response. Upon activation of a calcium permeable receptor, Ca^2+^ enters the cell and upon binding to TN-XXL produces a conformational change eliciting a fluorescence change reflected in quenching of the donor fluorescence and excitation of the acceptor.

For selection of pharmacologic leads, a facilitated and rapid assay of nAChR subtype selective agents is required early on in the screening process [10]. While electrophysiological methods through whole cell recording and patch clamp analysis can subsequently be used to uncover mechanism, they lack the scalability, cost reduction and automation that can be attained through the use of photon generated signals from multi-well plates [11], [12]. The scalability and automation derived from fluorometric imaging plate readers (FLIPR) have encouraged the use of fluorescence-based dyes in screening therapeutics on nAChRs [13], [14]. Yet conventional fluorescent dyes to detect depolarization or intracellular calcium, are limited by cost, variations in dye administration, dye shelf life, and cell perturbations during injection. Hence a stable, clonal cell line containing a light detection sensor and a receptor, which is generated from incorporating the respective genetic material, become critical considerations in developing a medium throughput assay for selective receptor activation. Bioluminescent Ca^2+^ reporters, such as GFP-aequorin, have been shown to be an applicable method in creating cell lines that contain a genetically encoded light detection method [15], [16]. Although bioluminescent reporters address the issues of variations in dye administration, shelf life, and cost; they are still susceptible to cell perturbations during injection, interference from fluorescent ligands, and are limited in time resolution due to the length of their decay. Applications that take advantage of fusing fluorescent protein Förster (or fluorescence) resonance energy transfer (FRET) pairs with Ca^2+^ binding proteins, such that changes in intracellular Ca^2+^ levels can be visualized through a combination of donor quenching and acceptor sensitization, have become a prevalent method for enabling investigators to monitor signal transduction pathways in varying cells [17]. The use of FRET pairs over Ca^2+^ sensing bioluminescent reporters minimizes the perturbations of ligand fluorescence and cell perturbation by solvent during the injection phase. Although many genetically encoded Ca^2+^ sensors exist [18], [19], the development of the Ca^2+^ sensor TN-XXL allowed for a highly sensitive and effective FRET based sensor [20]. Its incorporation with over-expressed G-protein-coupled receptors (GPCR) to produce cell-based neurotransmitter fluorescent engineered reporters (CNiFERs) offered the potential to overcome most of the limitations of the Ca^2+^ bioluminescent reporters [21]. In our previous studies, the generation of a genetically encoded sensitive Ca^2+^ sensor with a stably over-expressed GPCR allowed for *in vivo* validation of a compound's activity *in situ* in brain [21]. High-throughput fluorescence methodology [14], which has been previously utilized, affords a dual application to the stable, clonal cell line sensors we generated for transplanted cells. Here we report on generating and utilizing nAChR CNiFERs to measure nAChR activation via Ca^2+^ flux through ion channels in the cell membrane as opposed to GPCR mediated release primarily from intracellular stores described previously (Figure 1). Our studies extend the application of CNiFERs to prevalent and targeted CNS nAChR subtypes, the α7 and α4β2, and also the 5-HT_3A_ serotonin receptor. A chimaeric receptor CNiFER composed of α7/5-HT_3A_ was also generated to gain additional insight into activation of the wildtype α7-nAChR. The chimaeric receptor has been shown to have a desensitization rate comparable to 5-HT_3A_ receptors, and more recently a higher conducting variant has been created [22], [23], [24]. This chimaeric receptor still maintains ligand affinities for that of the full-length α7-nAChR, which allows for a positive allosteric modulator (PAM) free study of the α7-nAChR [23]. We report on a cell-based assay incorporating the use of nAChR CNiFERs to identify and characterize nAChR agonists and antagonists with a low cost, medium throughput fluorescence assay. The system is capable of detection on a monolayer of cells in a 7 mm diameter well of a 96-well plate and has the potential to be scaled to a high throughput platform.

## Results and Discussion

### Cell Based Neurotransmitter Fluorescent Engineered Reporters (CNiFERs)

**Figure 2:**
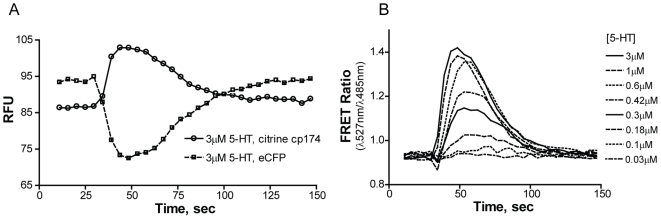
Direct FRET measurement of response. A. Measured time-course emission wavelengths for eCFP (485 nm) and Citrine cp174 (527 nm) on 5-HT_3A_ CNiFERs. 5-HT was injected at 30 s at 3 µM concentration to produce a Ca^2+^ influx causing a concentration dependent FRET response from the TN-XXL reporter. Measurements were taken every 3.52 s with excitation at 436 nm. B. FRET-Ratio (Citrine cp147/eCFP) time-course responses from 5-HT_3A_ CNiFERs to varying concentrations of 5-HT. Peak values or initial rates may be used to plot a concentration dependent curve to estimate EC_50_ values. Peak values were used to generate concentration response curves.

The first CNiFERs were developed for applications of monitoring cortical acetylcholine release in the cerebral cortex of rats using the M1-CNiFER [21]. The CNiFERs presented here were developed to facilitate pharmacological screening, potency rank ordering and characterization of compounds for calcium permeable ligand-gated ion channels (LGICs), with a particular focus on the homomeric α7 and heteromeric α4β2 nAChRs. The 5-HT_3A_ serotonergic LGIC receptor exists in its simplest form as a homopentamer similar to the α7-nAChR [25]. Although the 5-HT_3A_ receptor has a large calcium conductance, the 5-HT_3AB_ heteromeric receptors do not and therefore may have limited applicability with this technique [26]. Since antagonists, such as tropisetron, for the 5-HT_3A_ receptor are known to activate α7-nAChRs, 5-HT_3A_ CNiFERs not only expand the neurotransmitter target base, but also become a model system for testing opposing receptor responses [27]. LGIC agonist responses were measured with CNiFERs by monitoring TN-XXL FRET ratios, emissions of citrine cp174 (527 nm) to eCFP (485 nm), over 120 seconds with agonist injections occurring at the 30 second time-point. Figure 2a is an example of the fluorescent signals generated from a 5-HT_3A_ CNiFER single concentration response to 3 µM 5-Hydroxytryptamine (5-HT) injected at 30 seconds. FRET ratios of the emissions for a concentration range of 0.3–3 µM 5-HT is shown in Figure 2b. Concentration response curves can then be generated from the peak FRET ratio values for each concentration.

### α7-nAChR CNiFERs

**Figure 3:**
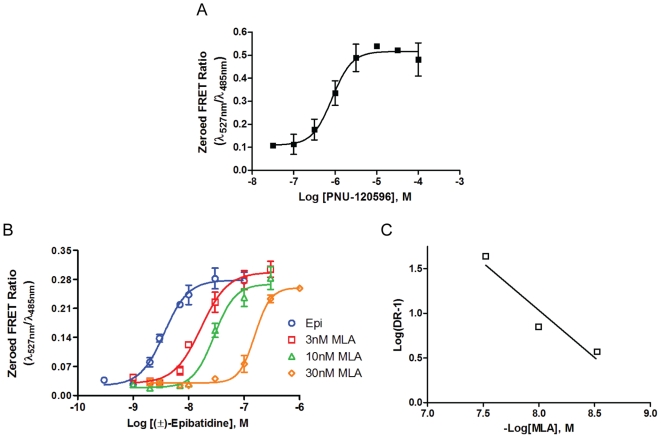
α7-nAChR CNiFER: PNU-120596 activation and methyllycaconitine antagonism. A. Concentration dependent response of PNU-120596 incubated for 30 minutes on α7-nAChR CNiFERs and stimulated with 0.1 µM (±)-epibatidine. The response curve shows that concentrations greater than 10 µM PNU-120596 would produce a maximal response. B. Competitive blockade of (±)-epibatidine elicited responses on α7-nAChR CNiFERs by MLA. The EC_50_ values for each curve shifted from 3.6 nM to 17 nM, 29 nM, and 160 nM for 3 nM, 10 nM, 30 nM MLA respectively. C. Schild plot of concentration dependent shifts. A slope of −1.2±0.3 confirmed MLA to block competitively.

Due to the rapid rate of desensitization of the α7-nAChR in the time frame of milliseconds [28], monitoring agonist-elicited fluorescence responses were not possible without including a PAM [29]. In our case we used the type II PAM, N-(5-Chloro-2,4-dimethoxyphenyl)-N'-(5-methyl-3-isoxazolyl)-urea (PNU-120596), and found that concentrations of 10 µM and above incubated on cells for at least 30 min (data not shown) yielded a maximal agonist-elicited fluorescence response (Figure 3a). Characteristic of a type II PAM, PNU-120596 will increase ion flux through the α7-nAChRs by decreasing its desensitization rate [30]. PNU-120596 likely interacts with a region in the transmembrane helices spanning TM1, TM2, and TM4 to prevent an activated α7-nAChR from switching to its desensitized state once activated [31]. PNU-120596 modulating activity results in an increase in the number of receptors remaining in an open or activated state. Accordingly, not only is a larger response produced, but also a lower concentration of agonist is likely needed to observe a response. Agonist EC_50_ values measured with the α7-nAChR CNiFER in the presence of PNU-120596 are less than those reported in the literature for α7-nAChR agonists without PNU-120596, as observed in Table 1. Nevertheless, these considerations are not likely to affect the rank ordering for agonist potencies. Assay of antagonists is conducted as a null method and should be independent of agonist used, provided a single receptor subtype is being analyzed. When (±)-epibatidine responses from α7-nAChR CNiFERs were blocked with three concentrations (3, 10, 30 nM) of methyllycaconitine (MLA) (Figure 3b). Schild analysis of the parallel concentration versus fluorescence readout showed MLA to block α7-nAChR CNiFERs competitively with a slope of −1.2±0.3 and with an observed K_a_ value of 3.2±1.4 nM for the antagonist (Figure 3c, Table 2).

**Table 1:** Comparison of Agonist EC_50_ using CNiFER and non-CNiFER detection^f^.

	α7/5-HT_3A_ CNiFER	α7/5-HT_3A_	α7-nAChR CNiFER	α7-nAChR	α4β2-nAChR CNiFER	α4β2-nAChR
**(**±**)-Epibatidine (EC_50_, nM)**	250±50 (n = 5)^a^	180±20^b^	28±16 (n = 24)^c^	17±2.6^d^	14±9 (n = 17)^a^	27±6 (n = 6)^d^
**(−)-Nicotine (EC_50_, nM)**	10,000±2,600 (n = 3)^a^	19,100±7,300^b^	410±270 (n = 10)^c^	1,160±180^d^ †, 92±12^e^	1,850±810 (n = 4)^a^	2,700±200^d*^,3,500^b*^

a. Measured in aCSF.

b. Measured with Ca^2+^ dye (Fluo-3-AM) on HEK293 cells expressing human receptors [23] *(Geometric Mean[-SD = 2.4, +SD = 5.1])[39].

c. Measured with 10 µM PNU-120596 in aCSF.

d. Measured with potential-sensitive dye against human receptors. *(2α3β) [35].

e. Measured with potential-sensitive dye and 3 µM PNU-120596.

† To be published Kuryatov A, Mukherjee J, and Lindstrom J.

f. Each n is calculated as an EC_50_ value from an individual concentration-response curve.

**Table 2:** Comparison of Antagonist K_a_ from CNiFERs to non-CNiFERs detection^f^.

LGIC-CNiFER	Antagonist	CNiFER K_a_, nM	Schild Slope	IC_50_, nM
**α7-nAChR**	MLA	3.2±1.4^a^ (n = 6)	−1.2±0.3	2.9±1.2^c^
**α7/5-HT_3A_**	MLA	2.5±0.1 b (n = 2)	−1.8±0.2	36±13^d^
**α4β2-nAChR**	DHβE	136±54 b (n = 5)	−0.9±0.1	88±24^c*^, 85(K_b_)^d*^
**5-HT_3A_**	Ondansetron	1.6±0.9 b (n = 7)	−1.3±0.04	16.0±5.7(K_i_)^e^

a. Measured with 10 µM PNU-120596 in aCSF.

b. Measured in aCSF.

c. Measured with potential-sensitive dye (to be published Kuryatov A, Mukherjee J, and Lindstrom J). *(2α3β) [40].

d. Measured with Ca^2+^ dye (Fluo-3-AM) on HEK293 cells expressing human receptors [23]. *(Geometric Mean [-SD = 66, +SD = 109])[39].

e. Measured competition against [^3^H]-ICs-205-930 in mouse neuroblastoma-glioma cells (NG-108-15) (5-HT_3AB_)[41], [42].

f. Each n is calculated from a Schild regression plot.

### α7/5-HT_3A_ Chimaeric Receptor CNiFER

**Figure 4:**
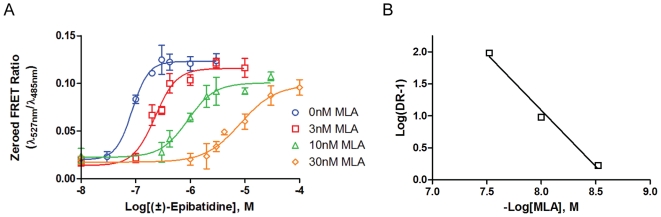
α7/5-HT_3A_ CNiFER: Methyllycaconitine antagonism. A. Competitive blockade of (±)-epibatidine elicited responses on α7/5-HT_3A_ CNiFERs by MLA. The EC_50_ values for each curve shifted from 86 nM to 230 nM, 900 nM, and 8.3 µM for 3 nM, 10 nM, 30 nM MLA respectively. B. Schild plot of concentration dependent shifts. A slope of −1.8±0.2 shows MLA to block competitively but with a percentage of receptors still occupied, attributing to a slight non-competitive component that may be an artifactual result of slow dissociation.

The chimaeric α7/5-HT_3A_ receptor does not exhibit the rapid desensitization rate seen with the wildtype (wt) α7-nAChR and thus neither requires PNU-120596, nor would PNU-120596 be effective [31], for achieving sufficient fluorescence signals within the time frame of the assay. In the presence of PNU-120596, agonists tend to have approximately a 10-fold higher affinity for the α7-nAChR (Table 1). The EC_50_ value for (±)-epibatidine measured in the presence of PNU-120596 for α7-nAChR CNiFERs was 28±16 nM, whereas the EC_50_ value measured without PNU-120596 on α7/5-HT_3A_ CNiFERs was 250±50 nM. In this case the chimaeric α7/5-HT_3A_ receptor can possibly yield measurements of potency closest to the actual channel opening event of the compound for the wt α7-nAChR. This difference may reflect affinity differences between the activatable and desensitized state of the receptor originally shown in the muscle nAChR [32]. Concentration-response curves generated with (±)-epibatidine on α7/5-HT_3A_ CNiFERs were blocked with MLA (3, 10, 30 nM) to characterize MLA antagonism. The resulting shifts were similar to those seen with the α7-nAChR CNiFERs (Figure 4a). The decrease in the maximum responses in the presence of MLA (Figure 4a) was also seen on several occasions with the α7-nAChR CNiFERs. This suggests a non-competitive component of antagonism, or that the half time of 2.3 min for dissociation of MLA is too slow to allow for complete dissociation from the receptor in the timeframe of our measurements [33]. Schild analysis showed a slope of −1.8±0.2, which indicates that MLA is not solely a competitive antagonist at the α7/5-HT_3A_ CNiFERs (Figure 4b). However, the major competitive component yielded a K_a_ of 2.5±0.1 nM, a value very close to that measured on the α7-nAChR CNiFERs (Table 2). The similarity of the antagonist affinities shows that using a PAM only affects agonist constants and employing the null method for determining K_a_ values negates the effect of PAMs. The similarity of these values for occupation by the antagonist validates the use of these chimaeric α7/5-HT_3A_ CNiFERs for characterizing ligands for the α7-nAChR without the need to use a PAM. Antagonist binding is more apt to be characterized by a single binding state as shown with the muscle nAChRs [34].

### α4β2-nAChR CNiFERs

**Figure 5:**
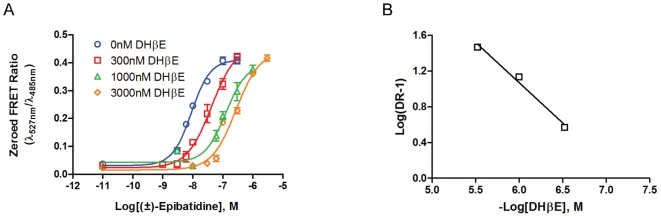
α4β2-nAChR CNiFER: DHβE antagonism. A. Competitive blockade of (±)-epibatidine elicited responses on α4β2-nAChR CNiFERs by DHβE. The EC_50_ values for each curve shifted from 8.9 nM to 42 nM, 130 nM, and 270 nM for 300 nM, 1 µM, 3 µM DHβE respectively. B. Schild plot of concentration dependent shifts. A slope of −0.9±0.1 confirmed DHβE to block competitively.

To characterize the α4β2-nAChR CNiFERs, concentration-response relationship data were generated with various concentrations of (±)-epibatidine in the absence and presence of a known competitive antagonist, dihydro-beta-erythroidine (DHβE) (0.3, 1, 3 µM). The resulting concentration-response curves showed a parallel shift with little decrease in the maximum revealing competitive antagonism (Figure 5a). Schild analysis confirmed DHβE acts competitively with a slope of −0.9±0.1 and measured a K_a_ value of 136±54 nM (Figure 5b, Table 2).

### 5-HT_3A_ Serotonin CNiFERs

**Figure 6:**
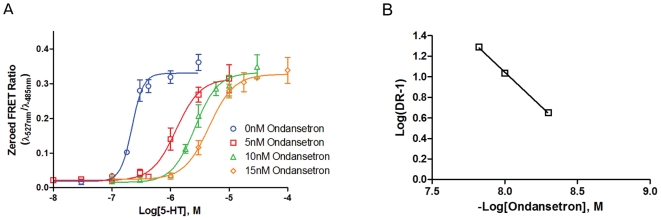
5-HT_3A_ CNiFER: Ondansetron antagonism. A. Competitive blockade of 5-HT elicited responses on 5-HT_3A_ CNiFERs by ondansetron. The steep Hill coefficient of 2.6 shows the more cooperative nature of the 5-HT_3A_ receptor. The EC_50_ values for each curve shifted from 220 nM to 1.6 µM, 2.6 µM, and 4.5 µM for 5 nM, 10 nM, 15 nM ondansetron respectively. B. Schild plot of concentration dependent shifts. A slope of -1.3±0.04 confirmed ondansetron to block competitively.

The 5-HT_3A_ receptor was generated from a single transfected subunit to form a homomeric pentamer. Concentration-response curves show serotonin to be an agonist, as expected, with an EC_50_ of 349±78 nM (n = 16). A near competitive block is achieved with ondansetron as an antagonist (Figure 6, Table 2). Other setrons, such as tropisetron and granisetron appear to show a greater non-competitive component (data not shown), but this observation may result from slow dissociation of the antagonist during the agonist exposure interval, as previously mentioned with MLA on the α7-nAChR.

### Concluding Analysis

CNiFER detection of agonist responses and blockade of the responses by antagonists, originally shown for a GPCR, muscarinic M1 receptor, are equally applicable to calcium permeable ligand-gated ion channels, as shown here for subunits of the Cys-loop receptor family. The chief advantages stem from the versatility of the system enabling one to measure responses to a variety of receptors in the same family by multi-array analysis. By developing the assay in a clonal cell line in which stable lines were first generated from the receptor subtype followed by plasmid transfection, or transduction with a viral vector containing the recombinant DNA encoded sensor, one can robustly generate previously established receptor subtype clones containing the FRET based sensor. These sensor clones may be used for either a robust medium-throughput assay or for *in vivo* implantation to determine neurotransmitter release [21]. The Molecular Devices FlexStation III instrument allows for multiple well samples in a 96 well plate to be assayed over 30 min plate intervals. This method could be scaled for use with higher throughput assaying via the FLIPR, or even for integration with high content screening platforms. Comparison of LGIC CNiFERs to their non-CNiFER counterparts shows similar ligand affinities and demonstrates that the TN-XXL sensor does not affect LGIC dissociation constants (Table 2). Our demonstration of (±)-epibatidine and 5-HT-elicited responses with predicted concentration ratio shifts from well known competitive antagonists provides a characterized cell system to distinguish defined receptor responses from indirect actions. We establish the utility of the CNiFER sensor for characterizing agonist and antagonist parameters for new ligand candidates on LGIC nicotinic and serotonin systems.

A chief limitation is the slow read time between measurements and the sensitivity of the sensor, resulting in difficulty with fast desensitizing receptor systems; yet with the judicious use of agents that maintain an active state and retard desensitization, this obstacle may be overcome and a suitable rank ordering of potency determined for agonists and antagonists. Distortions that might be achieved with more prolonged measurements of maximal depolarization can be minimized through monitoring the initial rate of depolarization. The slow off-rate antagonists that reduce signal and reveal an artifactual non-competitive component to antagonism exhibit another limitation from the reported on assay. By analyzing only the competitive aspect of the compound, one can effectively estimate K_a_ values. Therefore the use of LGIC CNiFERs in the development of therapeutics for CNS disorders is robust and cost-effective.

## Methods

### Generation of Stable Ca^2+^ Sensing nAChR Cell Lines

Individual HEKtsA201human α4β2 nicotinic receptor stable cell lines were generated as previously described [35]. Briefly human cDNA clones encoding α4 (pcDNA3.1/Zeo(−)) and β2 (pRc/CMV) nAChR subunits were transfected in equal amounts into HEKtsA201 cells using the FuGene6 transfection agent (Roche Diagnostics, Indianapolis, IN). Zeocin (0.5 mg/ml) was used for selection of α4 expression, and G418 (0.6 mg/ml; both from Invitrogen, Carlsbad, CA) was used for β2 selection to produce a stable, clonal cell line. Selection of the HEKtsA201 human α7-Ric3 nicotinic receptor stable cell lines and their pharmacological properties has been reported by Kuryatov, Mukherjee, and Lindstrom. The basic approach to preparing the line was similar to that used for the α4β2-nAChR cell line and its functional properties were similarly assayed using a potential-sensitive indicator after treatment with chemical chaperones to upregulate the amount of AChR. The stable clones were used to generate individual nAChR CNiFERs. The TN-XXL control CNiFERs (lacking over-expressed receptors) [20], [21], and mouse 5-HT_3A_ CNiFERs were developed as described in [21]. TN-XXL gene expression was introduced into each of the α7-Ric3 and α4β2 cells via lentiviral transduction as described [21] and fluorescence activated cell sorted (FACS) for high levels of eCFP and Citrine cp174 fluorescence. Chimaeric receptor DNA of human α7(1-202)/mouse 5-HT_3A_ was kindly provided by Dr. Steven Sine (Mayo Clinic, Rochester, MN). The α7/5-HT_3A_ chimaeric CNiFER was generated by calcium phosphate transfection of TN-XXL control cells with an α7/5-HT_3A_ gene subcloned into pcDNA3.1+ containing 5-HT_3A_ conductance-enhancing mutations of R432Q, R436D, and R440A [36]. Stable selection was achieved using G418 (0.5 mg/ml) to yield a TN-XXL control cell, as described in [21], containing over-expressed α7/5-HT_3A_ receptors, which were then selected by FACS as noted above.

### Flex Assay on Receptors

Cells were cultured in 10 cm plates with DMEM (Mediatech, Manassas, VA) supplemented with 10% FBS (Gemini Bio-Products, West Sacramento, CA; Atlanta Biologicals, Lawrenceville, GA) and 1% Glutamine (Invitrogen), and incubated at 37°C with 10% CO_2._ Cells were selected at ∼70% confluency and plated the day before using 100 µl volumes per well into black, transparent flat-bottom, TC treated 96-well plates (Thermo, Waltham, MA; E&K, Greiner Campbell, CA). Plates were removed from the incubator the next day and the media was replaced with 100 µl of artificial cerebral spinal fluid (aCSF) [containing 121 mM NaCl, 2.4 mM Ca^2+^, 1.3 mM Mg^2+^, 5 mM KCl, 26 mM NaHCO_3_, 1.2 mM Na_2_HPO_4_, 10 mM glucose, 5 mM Hepes Na^+^, pH 7.4]. For all assays performed on α7-nAChR CNiFERs 10 µM of the PAM, PNU-120596 (Tocris, Ellisville, MO), was prepared in aCSF. In the case of antagonist measurements, antagonists, at 1.5 times the final desired concentration, were prepared in aCSF. For non-CNiFER cell lines, Blue Membrane Potential dye (Molecular Devices, Sunnyvale, CA) was prepared in aCSF and diluted 2 fold the recommended dilution. Plates were incubated for at least 30 min at 37°C and 10% CO_2_ for any assay that included antagonist, dye, or PAM modification to the aCSF. Following incubation, plates were removed and placed into the Molecular Devices FlexStation III instrument and run at 37°C. Injection rates varied between 27–48 ml/s, and 50 µl of the specified agonist prepared at 3x final concentration in the aforementioned aCSF solution was injected after 30 s of baseline measurement. Reads of both donor eCFP (485 nm) and acceptor Citrine cp174 (527 nm) emission with eCFP excitation (436 nm) were made at 3.52 s intervals as shown in Figure 2a, or for non-CNiFER experiments with blue membrane dye, 565 nm was read with an excitation of 530 nm, for 90–120 s depending on the experiment. FRET ratios of the two wavelengths were plotted as shown in Figure 2b, and the peak height or initial rate of ion flux for the well was used to determine EC_50_ values for the injected agonist, acquired using SoftMax Pro 5.2 (Molecular Devices). All wells were replicated (two or three times) in the same plate. Mean peak FRET ratios calculated from replicates were exported and plotted against agonist concentrations using Graphpad Prism 4 (Carlsbad, CA). A sigmoidal concentration-response (variable slope) regression of the mean peak FRET ratios was fit to generate a concentration-response curve and obtain an EC_50_ value.

To measure potencies of competitive antagonists, three concentration-response curves with different antagonist concentrations were compared to a control curve (without antagonist). Mean FRET responses generated concentration response curves, and antagonist dependent shifts in EC_50_ values were used to calculate dose ratios (DR), as a concentration ratio of EC_50_'/EC_50_ for the specified agonist (where EC_50_' is in the presence of the antagonist at the specified concentration). DRs were used to produce a Schild regression plot for analysis of competitive antagonism and generate a K_a_ value. Analysis consisted of potting Log (DR-1) against –Log [Antagonist]. The Schild K_a_ value for competitive antagonists was calculated from the x-intercept at y = 0, as the –Log (K_a_) [37], [38]. All errors reported are arithmetic standard deviations unless otherwise noted.
